# Perioperative treatments for resected upper tract urothelial carcinoma: a network meta-analysis

**DOI:** 10.18632/oncotarget.12239

**Published:** 2016-09-24

**Authors:** Xiao Yang, Peng Li, Xiaheng Deng, Hongquan Dong, Yidong Cheng, Xiaolei Zhang, Chengdi Yang, Jingyuan Tang, Wenbo Yuan, Xiaoting Xu, Jun Tao, Pengchao Li, Haiwei Yang, Qiang Lu, Min Gu, Zengjun Wang

**Affiliations:** ^1^ Department of Urology, The First Affiliated Hospital of Nanjing Medical University, Nanjing, China; ^2^ Department of Anesthesiology, The First Affiliated Hospital of Nanjing Medical University, Nanjing, China

**Keywords:** upper tract urothelial carcinoma, perioperative treatment, network meta-analysis

## Abstract

**Background:**

Perioperative treatments have been used to improve prognosis in patients with upper tract urothelial carcinoma (UTUC). However, optimal management remains unestablished.

**Methods:**

We searched the Embase, Web of Science and Cochrane databases for studies published before June 20, 2015. All included studies were categorised into three groups on the basis of the outcome reported (overall survival (OS), disease-specific survival (DSS) and recurrence-free survival (RFS)). Relative hazard ratios (HRs) for death were calculated using random-effects Bayesian network meta-analysis methods. We also ranked the three different treatments in terms of three outcomes.

**Results:**

A total of 31 trials with 8100 patients were included. Compared with the control, adjuvant chemotherapy (AC) could improve OS, DSS and RFS by 32% (HR 0.68, 95% CI 0.51-0.89), 29% (HR 0.71, 95% CI 0.54-0.89) and 51% (HR 0.49, 95% CI 0.23-0.85), respectively. We noted a marked prolongation of RFS in both intravesical chemotherapy (HR 0.32, 95% CI 0.09-0.69) as well as concurrent radiotherapy and intravesical chemotherapy (HR 0.32, 95% CI 0.03-0.97) than in the control. Neoadjuvant chemotherapy (NAC) showed a significant improvement in DSS relative to the control (HR 0.25, 95% CI 0.06-0.61) and a distinct advantage over AC (HR 0.36, 95% CI 0.08-0.90) or AR (HR 6.89, 95% CI 1.25-18.66).

**Conclusions:**

Our results showed that AC; intravesical chemotherapy; and concurrent radiotherapy and intravesical chemotherapy could improve the prognosis of UTUC patients. NAC was found to be more favourable for UTUC than AC in terms of DSS.

## INTRODUCTION

Upper tract urothelial carcinoma (UTUC) is an uncommon cause of tumours worldwide; it accounts for 5% of urothelial cancers and 7%–8% of all renal tumours [1]. The disease often results in worse prognosis than renal cell carcinoma. Radical nephroureterectomy (RNU) is the gold standard treatment for UTUC patients [2, 3]. However, relapse in patients who underwent RNU is highly common (28%–61.4%), with a five-year recurrence-free survival (RFS) rate of 69% [4, 5]. As a consequence, perioperative treatment has been logically employed to reduce relapse and prolong survival. Major perioperative treatments include adjuvant radiotherapy (AR), adjuvant chemotherapy (AC), neoadjuvant chemotherapy (NAC), concurrent chemoradiotherapy and intravesical chemotherapy. However, optimum perioperative therapy remains inconclusive.

AC is the most widely used treatment in patients with cancer after undergoing surgery. Leow, J. J. et al. suggested the potential benefit in overall survival (OS) and disease-free survival (DFS) of cisplatin-based AC in UTUC [6]. In contrast to AC, NAC utilises agents to reduce the burden of tumour before patients undergo surgical treatment. A retrospective review found that NAC may prolong the survival of patients compared with the matched cohort who underwent initial surgery, but additional trials are necessary to confirm the treatment's utility [7]. The role of AR for transitional cell carcinoma (TCC) also remains controversial [8]. Maulard-Durdux et al. [9] found that the local control of disease and survival were similar in both treatment and control groups. However, some other trials showed that radiotherapy may improve overall patient survival [10, 11]. A previous meta-analysis comparing adjuvant radiation therapy with concurrent chemoradiotherapy revealed that concurrent chemoradiotherapy could improve the outcomes of patients with resected locally advanced upper tract urothelial malignancies [12]. Intravesical chemotherapy is intended to reduce the recurrence rate in UTUC patients who underwent RNU. Several studies [13-15] concluded that intravesical chemotherapy may significantly reduce the bladder malignancy recurrence rate and prolong the time to first bladder recurrence, which suggest the utility of intravesical chemotherapy in UTUC patients.

In this study, we aimed to assess the prognosis of UTUC patients who received different treatment strategies after RUN. When direct comparisons are unavailable, investigators can use Bayesian network meta-analysis to assess the efficacy of different treatment strategies indirectly. To establish the optimal treatment for UTUC patients who underwent RNU accompanied with removal of an ipsilateral bladder cuff, we conducted a network meta-analysis to evaluate the efficacy of major perioperative treatments, including AR, AC, NAC, concurrent chemoradiotherapy and intravesical chemotherapy.

## MATERIALS AND METHODS

### Search strategy and selection criteria

**Figure 1:**
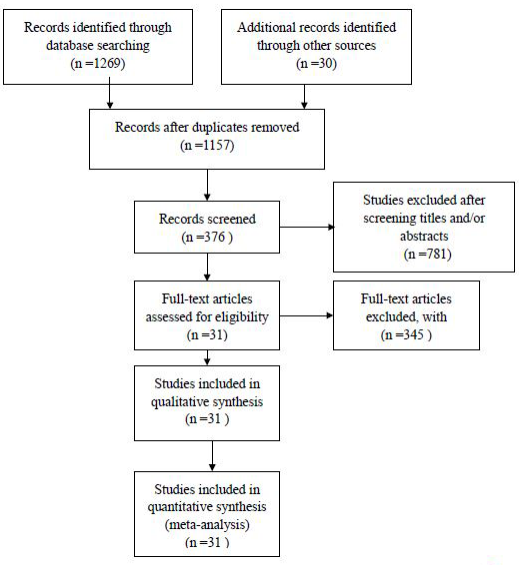
Literature search and selection

We searched PubMed, the Cochrane Collaboration Central Register of Controlled Clinical Trials, Web of Science and Embase for eligible studies. In the process, we used the keywords “upper tract urothelial carcinoma [Title/Abstract] AND (chemotherapy [Title/Abstract] OR radiotherapy [Title/Abstract] OR intravesical chemotherapy [Title/Abstract])” and other related words to search for studies published until the end of June 20, 2015. We did not impose any restriction on the publication date or publication status. The search strategies are summarised in Figure 1. We also searched for additional relevant studies by browsing the bibliographies of the included trials and related reviews.

We included case–control studies that compared at least one of the five perioperative treatments described above with placebo or with another treatment involved. We chose the latest updated data for analysis if two or more studies report the same trials at different follow-up periods. We excluded the studies that contained none of the five treatment strategies. To ensure the accuracy and repeatability of our study, two researchers reviewed all of the eligible studies to determine which studies satisfied the inclusion criteria. Conflicts were resolved by a third reviewer.

### Data extraction and synthesis

Baseline characteristics, selection criteria, treatment strategies and outcomes were extracted independently by two investigators. Any disagreement was resolved by a third investigator. We focused on the following outcomes: DSS, OS and RFS(Including local, intravesical and contralateral upper tract recurrence).

We divided all of the studies into three groups on the basis of the three outcomes described above. One study would be classified under different groups if two or more outcomes were reported by the work.

We considered the hazard ratio as our outcome measure because this parameter can provide intuitive comparison of different treatments and is often adjusted for confounders. When HRs were not reported in the studies, we obtain estimates of the value on the basis of the data provided in the manuscripts under study as we described previously [16].

### Statistical analysis

The pooled HR with corresponding 95% confidence interval (CI) was calculated in accordance with Tierney's method [17]. Bayesian network meta-analysis was performed with WinBUGS version 1.4.3 (MRC Biostatistics Unit, Cambridge, UK). We used an indirect comparison model to calculate the HRs in two incomparable heal methods. After setting randomised starting values, we used 100000 times simulated annealing, yielding 200000 iterations (100000 per chain) to obtain the HR of model parameters. The convergence of iterations was assessed with Gelman–Rubin–Brooks statistic [18].

### Quality evaluation

Quality evaluation of each included study was performed utilising the Newcastle–Ottawa Scale (NOS) [19]. The assessment consisted of three major categories: selection (four items, one star for each), comparability (one item, up to two stars) and exposure (three items, one star for each). A maximum of nine stars can be given to one study. A final score of six stars or more was regarded as high quality.

## RESULTS

The titles and abstracts of 1157 potentially relevant articles were reviewed for initial screening (Figure 1). We then retrieved the full text of potentially eligible articles for detailed information. A total of 31 articles (Table 1) satisfied the inclusion criteria and were included in the network meta-analysis, with 8100 patients receiving at least one of the five treatments. All of the included studies had been published and were available in full manuscripts.

**Table 1:** Characteristics of the studies included in the meta-analysis

Author	Year	Country	Number	Stage	Intervention	Disease specific survival		Overall survival		Recurrence free survival		Follow-up (months)	
						HR	95%CI	HR	95%CI	HR	95%CI	Median	range
Hall MC [32]	1998	American	74	3-4	AR vs. ctl AR vs. ctl	0.98 0.87	[0.42,2.25] [0.32,2.36]					21	[1,236]
Lee S. E. [37]	2006	Korea	27	3	AC vs. ctl	0.62	[0.18,2.17]					48	[25,102]
Seitz C. [38]	2010	Spain+Italy+American+Japan+Canada+Germany	754	1-4	AC vs. ctl	0.40	[0.24,0.63]			0.42	[0.27,0.67]	40	[18,75]
Kawashima A [39]	2012	Japan	93	3	AC vs. ctl	0.21	[0.06,0.66]						
Yafi F. A [20].	2014	Canada	305	1-4	AC vs. ctl	0.78	[0.40,1.50]	0.70	[0.29,1.66]			17.8	[5.5,46.8]
Kluth L. A. [40]	2013	Germany+France+Japan+Austria+Italy+American	242	1-4	AC vs. ctl AC vs. ctl	1.57 0.87	[0.86,2.84] [0.60,1.25]					9	
Raman J. D. [41]	2014	France+American	414	0-4	AC vs. ctl	0.85	[0.71,0.95]					16	[2,120]
Kim T. S [42].	2013	Korea	65	1-4	AC vs. ctl	0.52	[0.17,1.82]			0.07	[0.01,0.31]	34	[12,114]
Porten S. [7]	2014	American	112	0-4	NAC vs. ctl	0.19	[0.06,0.61]						
Huang [22]	2015	ChinaTaiwan+USA	171	1-4	AC vs. ctl	0.60	[0.34,1.05]	0.51	[0.34,0.74]	0.61	[0.45,0.83]	35.8	[3.4,125.2]
Lee [21]	2015	Korea	324	1-4	AC vs. ctl	0.74	[0.49,1.13]	0.74	[0.49,1.11]			53.9	[1,297]
Kuriyama M [43]	1987	Japan	37	1-3	AC vs. ctl			1.37	[0.49,3.85]	0.56	[0.10,3.12]		
Cozad SC [44]	1992	American	26	3	AR vs. ctl			0.55	[0.24,1.25]	0.19	[0.01,2.63]	13.5	[3,311]
Sengeløv L [23]	1994	Denmark	240	1-4	AC vs. ctl			0.41	[0.30,0.57]				
Suzuki S [45]	2004	Japan	56	1-4	AC vs. ctl			0.60	[0.25,1.45]			39	[4,163]
Czito B [12]	2004	American	31	1-4	AR vs. radio			0.47	[0.14,1.64]			31.2	[1.2,224.4]
Kwak C [46]	2006	South Korea	43	2-3	AC vs. ctl			0.11	[0.02,0.53]			30.7	[4.7,98.8]
Soga N [47]	2008	Japan	46	2-3	AC vs. ctl			0.68	[0.09,5.21]	0.14	[0.04,0.58]		
Hellenthal N. J. [48]	2009	Austria+American+Italy+Germany+Canada+France+Japan	542	3-4	AC vs. ctl			1.06	[0.80,1.40]			26	[0,231]
Chen B [11]	2011	China	133	1-4	radio+intravesical vs. intravesical			0.80	[0.43,1.50]	0.48	[0.24,0.98]	26.6	
Fan KH [49]	2012	China Taiwan	40	0,3-4	AC vs. ctl			1.01	[0.28,3.57]			61	[22,93]
Kitamura H [50]	2012	Japan	29	1-4	NAC vs. ctl			0.38	[0.15,0.94]			81	[19,201]
Jwa E [10]	2014	Korea	127	1-4	AC vs. ctl AR vs. ctl			0.91 0.98	[0.52,1.58] [0.51,1.90]			38.3	[7.3,154.3]
Cozad SC [51]	1995	American	67	1-4	AR vs. ctl					0.23	[0.01,3.71]		
Sakamoto N [52]	2001	Japan	25	1-2	postoperative instillation vs. ctl					0.12	[0.01,1.07]	45	[6,65]
Wu W. J. [13]	2010	China Taiwan	196	1-3	postoperative instillation vs. ctl postoperative instillation vs. ctl					1.88 0.95	[0.89,3.95] [0.47,1.93]		[12,182]
Vassilakopoulou M [26]	2011	Greece+France	627	3-4	AC vs. ctl AC vs. ctl					1.43 1.10	[0.52,3.85] [0.83,1.47]	22.5	[10,50]
O'Brien T [14]	2011	United Kingdom	239	1-4	postoperative instillation vs. ctl					0.66	[0.35,1.28]		
Ito A(160) [35]	2013	Japan	72	1-3	AC vs. ctl postoperative instillation vs. ctl					1.13 0.26	[0.30,4.19] [0.07,0.91]		
Ito A(70) [15]	2013	Japan	72	1-3	AC vs. ctl postoperative instillation vs. ctl radio+intravesical vs. intravesical					1.16 0.02 6.45	[0.18,7.37] [0.01,0.53] [0.50,83.2]		
Shirotake [53]	2015	Japan	839	1-4	AC vs. ctl					1.22	[1.02,1.44]	32	[16,62]

Abbreviations: AR: adjuvant radiotherapy, AC: adjuvant chemotherapy, NAC: neoadjuvant chemotherapy

### OS analysis

**Figure 2:**
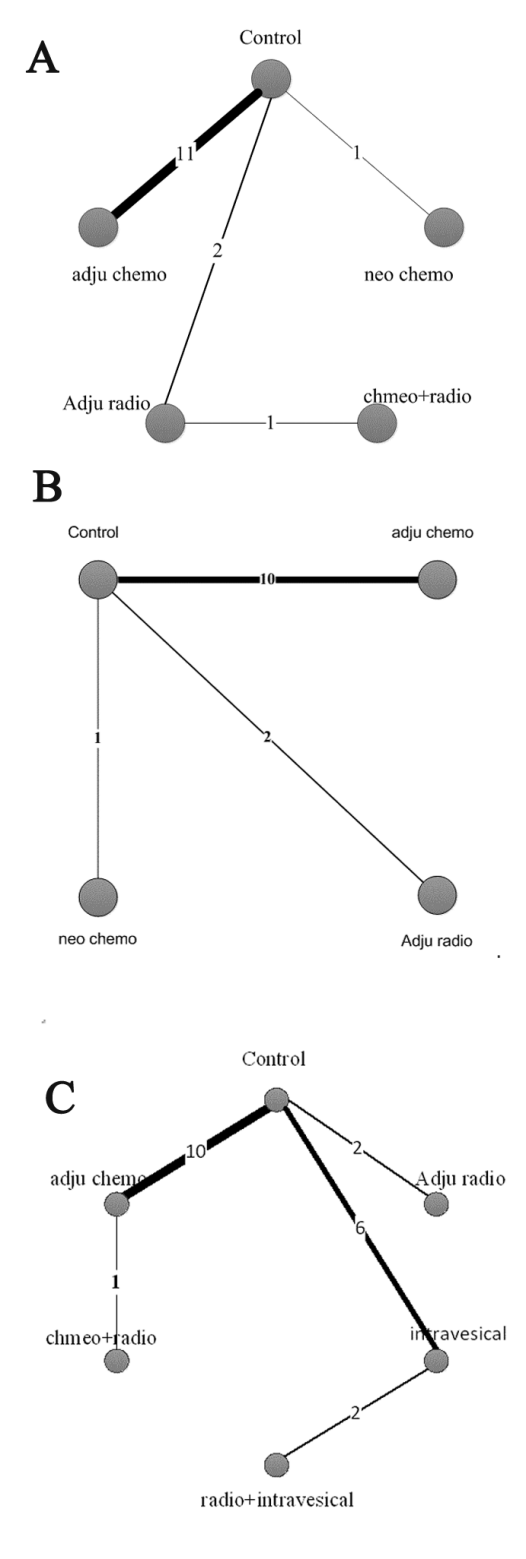
Network of comparisons of OS, DSS and RFS for Bayesian network meta-analysis The width of each line is proportional to the number of trials (on the line) compared with the connected treatments. A OS, B DSS and C RFS.

**Figure 3:**
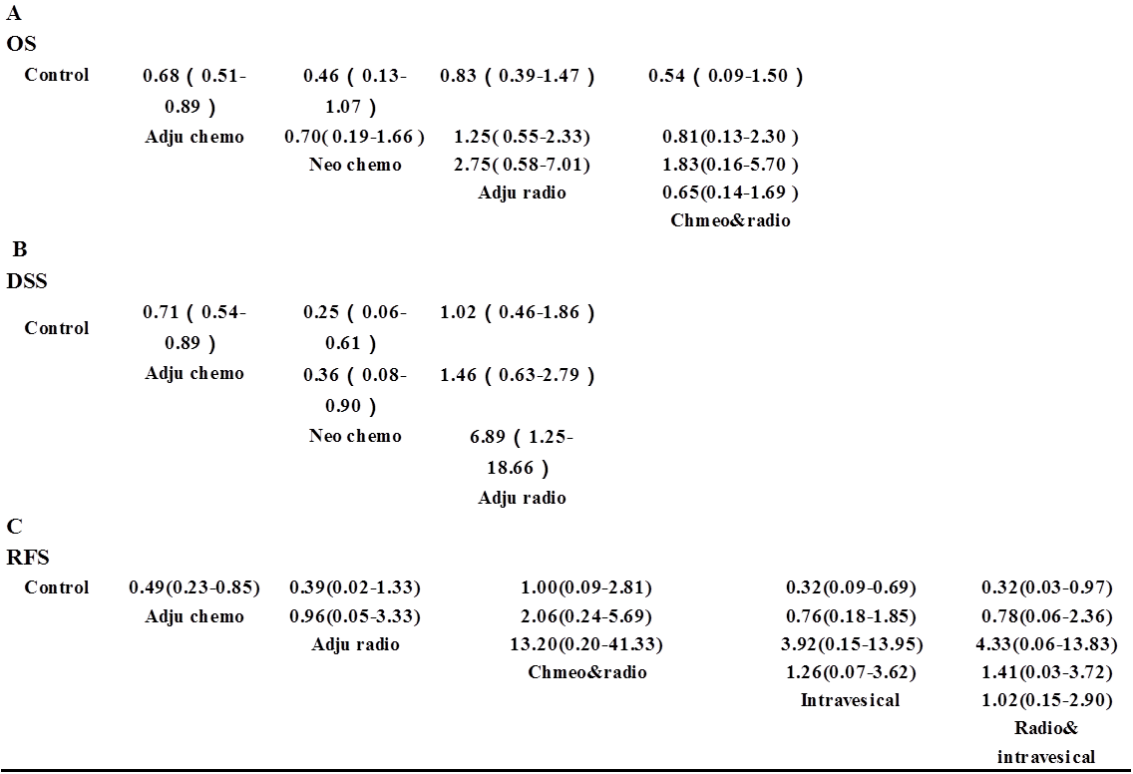
Pooled HRs for OS (A), DSS (B) and RFS (C) The column treatment is compared with the row treatment. HRs with Bayesian p values less than 0.05 are indicated in green and those rescued are in red.

Of the 31 included articles, 15 studies with a total of 2150 patients reported OS and were placed into the OS subgroup. Four treatments were adopted in the involved trials (Figure 2A). HRs were reported in all 11 trials. The results of our random-effects network meta-analysis for OS are summarised in Figure 3A. We find that, compared with control, AC improved patient OS by 32% (HR 0.68, 95% CI 0.51–0.89). The trend of prolonged OS was also observed with NAC, but the effect was not significant (HR 0.46, 95% CI 0.13–1.07). Meanwhile, the data showed no significant prolonging of OS with AR (HR 0.83, 95% CI 0.39–1.47) and concurrent chemoradiotherapy (HR 0.54, 95% CI 0.09–1.50). Furthermore, no significant improvement was observed between different treatment strategies (Figure 3A).

**Figure 4:**
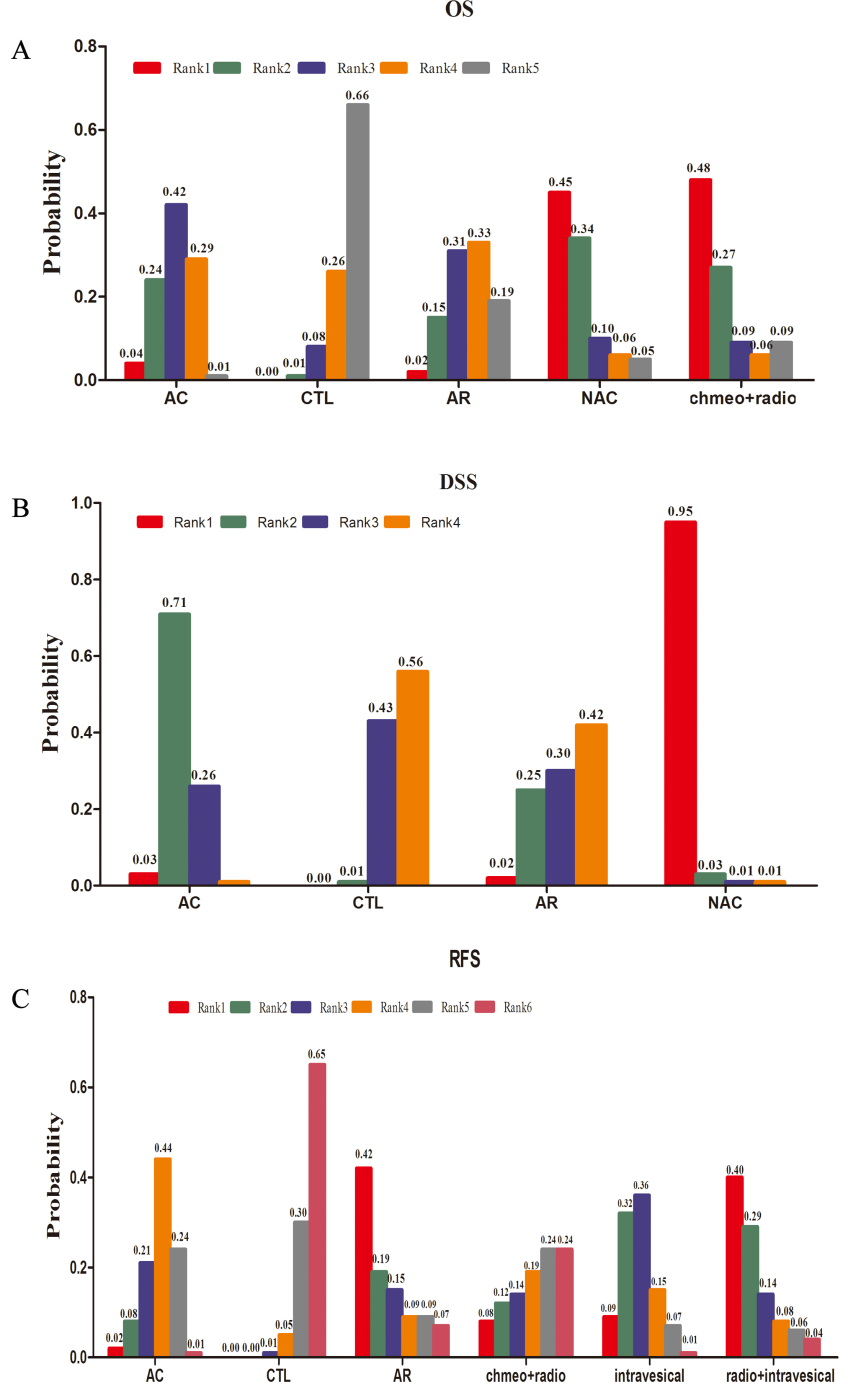
Values of the different rankings of the treatment strategies A OS, B DSS and C RFS

In Figure 4A, we summarised the possibility value (PV) of the different rankings of each treatment strategy. The control was most likely to be ranked with the shortest OS (PV=0.66). AR was ranked with the second shortest OS (PV=0.33), followed by AC (PV=0.42). The data showed that NAC (PV=0.45) and concurrent chemoradiotherapy (PV=0.48) were both most likely to be ranked with the longest OS.

### DSS analysis

A total of 2581 patients with three treatment strategies were included in the DSS group (Figure 2B). We summarised the results of the random-effects network meta-analysis for DSS in Figure 3B. Compared with the control, AC and NAC markedly prolonged DSS by 29% (HR 0.71, 95% CI 0.54–0.89) and 75% (HR 0.25, 95% CI 0.06–0.61), respectively. By contrast, AR showed no significant improvement in DSS relative to the control (HR 1.02, 95% CI 0.46–1.86). Notably, we observed a distinct improvement in DSS with NAC relative to AC (HR 0.36, 95% CI 0.08–0.90). Moreover, AR showed poor DSS when compared with AC (HR 6.89, 95% CI 1.25–18.66). No statistically significant difference was observed between AR and AC (HR 1.46, 95% CI 0.63–2.79).

The ranking of these three treatment strategies and control are displayed in Figure 4B. NAC (PV=0.95) or AC (PV=0.71) were most likely ranked with longest or second-longest DSS. In addition, the control (PV=0.56) and AR (PV=0.42) were both ranked as the poorest treatment strategies for DSS.

### RFS analysis

A total of 15 studies with 3369 patients were included in the RFS subgroup (Figure 1). As the 15 studies didn’t describe their definitions of recurrence definitely, we made a preminary analysis without separating local recurrence from intravesical recurrence and contralateral upper tract recurrence. The network of five treatment strategies and control is displayed in Figure 2C.

Compared with the control group, AC, intravesical chemotherapy and concurrent chemoradiotherapy were noted to improve the RFS (HR 0.49, 95% CI 0.23–0.85 for AC, HR 0.32, 95% CI 0.09–0.69 for intravesical chemotherapy and HR 0.32, 95% CI 0.03–0.97 for concurrent radiotherapy and intravesical chemotherapy). Data showed no significant difference between the various treatment strategies.

In Figure 4C, we summarised the values of the different rankings of the five treatment strategies and control. The control was most likely to be ranked with the shortest RFS (PV=0.65). In contrast to the control, both AR (PV=0.42) and concurrent radiotherapy and intravesical chemotherapy (PV=0.40) were ranked with the highest RFS. Intravesical chemotherapy (PV=0.36), AC (PV=0.44) and concurrent chemoradiotherapy (PV=0.24) were most likely to be ranked as third, fourth and fifth, respectively.

### Quality assessment

The results of quality assessment for the included studies using NOS are shown in Table 2. The scores range from five to seven stars; 28 studies scored six stars or more, indicating moderate or high quality.

**Table 2:** Results of quality assessment by NOS

Study	1	2	3	4	5A	5B	6	7	8	Scores
Hall MC 1998	*	*	-	-	*	-	*	*	-	5
Lee S. E. 2006	*	*	-	*	*	*	*	*	-	7
Seitz C. 2010	*	*	-	*	*	*	*	*	-	7
Kawashima A 2012	*	*	-	*	*	*	*	*	-	7
Yafi F. A 2014	*	*	-	*	*	*	*	*	-	7
Kluth L. A. 2013	*	*	-	*	*	*	*	*	-	7
Raman J. D. 2014	*	*	-	*	*	*	*	*	-	7
Kim T. S 2013	*	*	-	*	*	*	*	*	-	7
Porten S. 2014	*	*	-	*	-	*	*	*	-	6
Huang 2015	*	*	-	*	*	*	*	*	-	7
Lee 2015	*	*	-	*	*	*	*	*	-	7
Kuriyama M 1987	*	*	-	*	-	-	*	*	-	5
Cozad SC 1992	*	*	-	*	*	-	*	*	-	6
Sengeløv L 1994	*	*	-	*	*	*	*	*	-	7
Suzuki 2004	*	*	-	*	-	*	*	*	-	6
Czito B 2004	*	*	-	*	*	-	*	*	-	6
Kwak C 2006	*	*	-	*	*	*	*	*	-	7
Soga N 2008	*	*	-	*	-	-	*	*	-	5
Hellenthal N. J. 2009	*	*	-	-	*	*	*	*	-	6
Chen B 2011	*	*	-	*	*	*	*	*	-	7
Fan KH 2012	*	*	-	*	*	*	*	*	-	7
Kitamura H 2012	*	*	-	-	*	*	*	*	-	6
Jwa E 2014	*	*	-	*	*	*	*	*	-	7
Cozad SC 1995	*	*	-	*	-	*	*	*	-	6
Sakamoto N 2001	*	*	-	*	*	*	*	*	-	7
Wu W. J 2010	*	*	-	*	*	*	*	*	-	7
Vassilakopoulou M 2011	*	*	-	*	*	*	*	*	-	7
O'Brien T 2011	*	*	-	*	*	-	*	*	-	6
Ito A(160) 2013	*	*	-	*	*	*	*	*	-	7
Ito A(70) 2013	*	*	-	*	*	*	*	*	-	7
Shirotake 2015	*	*	-	*	*	*	*	*	-	7

1 indicates adequate definition of cases; 2 cases are representative of the population; 3 community controls; 4 controls have no history of UTUC; 5 study controls for tumour stage and grade; 5B study controls for additional factor(s); 6 ascertainment of exposure by blinded interview or record; 7 same method of ascertainment used for cases and controls; 8 similar non-response rates for cases and controls.

## DISCUSSION

To date, RNU remains as the gold standard treatment for high-risk UTUC. However, relapse and metastasis are highly common in UTUC patients after RNU, affecting long-term survival. Perioperative treatments have been used to reduce relapse and prolong survival. However, the optimal perioperative therapy is still uncertain.

Our network meta-analysis is the first study assessing different peri-surgical treatment strategies for UTUC. Our results suggest that both AC and NAC are advantageous over the control in terms of OS and DSS. Furthermore, AC prolongs the RFS by 51%, whereas NAC exhibits no significant improvement of RFS. The prolongation of RFS is also observed in the patients who received intravesical chemotherapy or concurrent radiotherapy and intravesical chemotherapy. In terms of DSS, NAC shows better prognosis than AC and AR.

Controversial results on the effect of AC have been reported in the management of patients receiving RNU [20-23]. A definite consensus on this issue will unlikely be achieved until further evidence from an ongoing prospective trial is available^ [24]^. Even so, our pooled results suggest that AC can improve OS by 32% relative to the control (HR 0.68, 95% CI 0.51–0.89). In terms of DSS, the improvement is also remarkable (HR 0.71, 95% CI 0.54–0.89). For RFS, prolongation can be seen in 51% of the patients (HR 0.49, 95% CI 0.23–0.85). AC application after surgery shows many advantages. Through this strategy, we can assess the pathologic staging immediately, which means the absence of delay in treatment for high-risk patients and the reservation of local surgical treatment for non-responders [25]. Moreover, utilisation of AC can destroy the tumour cells that are locally implanted, retained or present in the blood cycle to form micrometastasis before or during surgical operation. These reasons may explain why AC prolongs patient survival by 51% than the control in our study.

Previous studies also reported conflicting outcomes in patients who received NAC [26-28]. In our study, NAC demonstrates a considerable beneficial effect on DSS (HR 0.25, 95% CI 0.06–0.61). The HRs were 0.36 for the indirect comparison of NAC with AC and 6.89 for the comparison between AR and NAC, suggesting that NAC holds an obvious advantage on improving patient DSS. Survival benefit is also observed in terms of OS (HR 0.46, 95% CI 0.13–1.07) in our study. NAC exhibited some drawbacks, including overtreatment of non-responders and impairment of renal and hepatic function. Nevertheless, patients could still benefit from NAC. For instance, NAC may cure micrometastatic disease, reduce the circle tumour cells and prolong survival. Several studies reported that NAC could prolong the survival of patients with bladder cancer [29], improve the complete response rate and downstaging rate in UTUC patients [27]. These findings support the use of NAC in UTUC patients. The contemporary view is that NAC should be better than AC. Large prospective RCTs are needed to determine whether this is true in UTUC.

For most common cancers, AR is essential to control local–regional recurrence, palliate symptoms and manage localised disease [30]. In UTUC patients, the rate of local–regional recurrence may reach up to 45% [31]. Thus, the role of AR must be addressed. Controversial results on the effectiveness of AR were reported in previous studies [11, 12, 32]. In our study, AR demonstrates no beneficial effect for the improvement of OS (HR 0.83, 95% CI 0.39–1.47), DSS (HR 1.02, 95% CI 0.46–1.86) and RFS (HR 0.39, 95% CI 0.02–1.33). This finding may be due to the fact that the patients included in previous studies involved different clinical stages and pathological grades that we cannot adjust. Furthermore, the relatively small number of patients in our study also limits our accuracy in assessing the role of AR. Our data indicate that concurrent chemoradiotherapy shows no survival benefit in terms of OS and RFS, which contradicts the findings from previous studies [12, 26]. By contrast, utilisation of AC alone can improve both OS and RFS, as shown in Figure 3. This result may be attributed to the side effects of radiotherapy. Furthermore, UTUC patients are usually elderly [24] who are underrepresented in oncology trials [33], suggesting that they were at higher risk of side effect of radiotherapy. Our results are consistent with the 2015 EAU guideline that radiotherapy is no longer relevant, either alone or as adjunct to chemotherapy [24].

Two independent prospective randomised controlled trials demonstrated that postoperative instillation of intravesical mitomycin C with or without cytosine arabinoside reduces the risk of recurrence of bladder tumours after surgery for UTUCs [14, 34]. This treatment was confirmed in other prospective randomised trials to be replaced by pirarubicin [13, 35] and in a meta-analysis [34]. In our study, intravesical chemotherapy (HR 0.32, 95% CI 0.09–0.69) is noted to prolong RFS by 68%. Interestingly, the HR of concurrent radiotherapy and intravesical chemotherapy is 0.32, and concurrent radiotherapy plus intravesical chemotherapy is not better than intravesical chemotherapy alone (HR 1.02, 95% CI 0.15–2.90). This finding may be attributed to the conclusion discussed above that radiotherapy is no longer relevant, either alone or as an adjunct to chemotherapy [24]. Our work demonstrates that patients should receive prophylactic intravesical chemotherapy after nephroureterectomy to reduce recurrence.

Several limitations exist in our study. First, we analysed data from previous trials rather than individual patient data. Similar covariates, which might affect the final outcomes, are present at the individual patient level, which we cannot adjust. Secondly, for the analysis of NAC versus AC, only one study was included due to the limited number of published studies which might weaken the conclusion.Another limitation is that RNU was performed by various surgeons across different trials over a long period, which might influence survival to a large extent. Additionally, significant heterogeneity exists among the procedures adopted in the studies. For example, different trials exploring AC varies in the regimen, dosage and number of cycles. Nevertheless, our study also offered several advantages as follows. When direct comparisons were unavailable, our network meta-analysis assessed the efficacy of different treatment strategies. This meta-analysis can combine direct and indirect comparisons to strengthen the efficiency and reliability of the study. The measures of survival also varied among studies. Our meta-analysis overcame this disadvantage through a single analysis using the HR scale, thereby avoiding potential selection bias and loss of information from only including studies with the same measure or performing separate analyses for different measures [36]. Our work synthesised all available existing evidence and provided new evidence on controversial issues, presenting important implications in clinical practice and future research.

In conclusion, our results suggest that both AC and NAC improve OS and DFS of UTUC patients after RNU. Furthermore, AR is found to no longer be relevant for use either alone or as an adjunct to chemotherapy. Finally, AC, intravesical chemotherapy or concurrent radiotherapy and intravesical chemotherapy prolong RFS.
